# Mid-Infrared Imaging Characterization to Differentiate Lung Cancer Subtypes

**DOI:** 10.3389/pore.2022.1610439

**Published:** 2022-08-17

**Authors:** E. Kontsek, A. Pesti, J. Slezsák, P. Gordon, T. Tornóczki, G. Smuk, S. Gergely, A. Kiss

**Affiliations:** ^1^ 2nd Department of Pathology, Semmelweis University, Budapest, Hungary; ^2^ Department of Applied Biotechnology and Food Science, Budapest University of Technology and Economics, Budapest, Hungary; ^3^ Department of Electronics Technology, Budapest University of Technology and Economics, Budapest, Hungary; ^4^ Department of Pathology, Medical School and Clinical Center, University of Pécs, Pécs, Hungary

**Keywords:** lung cancer, fingerprint region, transflectance, SVM, LDA, FTIR, infrared

## Abstract

**Introduction:** Lung cancer is the most common malignancy worldwide. Squamous cell carcinoma (SQ) and adenocarcinoma (LUAD) are the two most frequent histological subtypes. Small cell carcinoma (SCLC) subtype has the worst prognosis. Differential diagnosis is essential for proper oncological treatment. Life science associated mid- and near-infrared based microscopic techniques have been developed exponentially, especially in the past decade. Vibrational spectroscopy is a potential non-destructive approach to investigate malignancies.

**Aims:** Our goal was to differentiate lung cancer subtypes by their label-free mid-infrared spectra using supervised multivariate analyses.

**Material and Methods:** Formalin-fixed paraffin-embedded (FFPE) samples were selected from the archives. Three subtypes were selected for each group: 10-10 cases SQ, LUAD and SCLC. 2 μm thick sections were cut and laid on aluminium coated glass slides. Transflection optical setup was applied on Perkin-Elmer infrared microscope. 250 × 600 μm areas were imaged and the so-called mid-infrared fingerprint region (1800-648cm^−1^) was further analysed with linear discriminant analysis (LDA) and support vector machine (SVM) methods.

**Results:** Both “patient-based” and “pixel-based” approaches were examined. Patient-based analysis by using 3 LDA models and 2 SVM models resulted in different separations. The higher the cut-off value the lower is the accuracy. The linear C-support vector classification (C-SVC) SVM resulted in the best (100%) accuracy for the three subtypes using a 50% cut-off value. The pixel-based analysis gave, similarly, the linear C-SVC SVM model to be the most efficient in the statistical indicators (SQ sensitivity 81.65%, LUAD sensitivity 82.89% and SCLC sensitivity 88.89%). The spectra cut-off, the kernel function and the algorithm function influence the accuracy.

**Conclusion:** Mid-Infrared imaging could be used to differentiate FFPE lung cancer subtypes. Supervised multivariate tools are promising to accurately separate lung tumor subtypes. The long-term perspective is to develop a spectroscopy-based diagnostic tool, revolutionizing medical differential diagnostics, especially cancer identification.

## Introduction

### Lung Tumors

In 2018 lung cancer was the most commonly diagnosed tumor and the leading cause of death in both sexes worldwide [1]. Preoperative biopsy materials have particular importance and the volume of biopsy material is limited. The clinical sampling also determines the feasible pathological methods. Brush cytology is often performed, however, diagnostic methods on cells or cell groups are more limited than histological analysis on FFPE samples collected by bronchoscopic tissue sampling. There is a need for ancillary diagnostics to determine histological subtypes to save material for the upcoming molecular diagnostics. Infrared spectroscopy might be one of these tools [2]. Optical fibre-based techniques combined with bronchoscopes or transthoracic needles may also disrupt and improve the current diagnostic pathways [3].

For a long time, the differential diagnosis between small cell versus non-small cell lung cancer was the most important clinicopathological aspect. Squamous cell carcinoma (SQ) and adenocarcinoma (LUAD) are the two most frequent histological subtypes. Small cell carcinoma (SCLC) subtype has the worst prognosis. There are rare tumor types or mixed entities such as mesothelioma or adenosquamous carcinoma. The development of targeted therapies and their spread in the routine oncological treatment required subtype-specific differentiation and definition of the tissue of origin even in dedifferentiated tumors and cytological specimens. This can be achieved by immunohistochemical typing [4, 5]. The most targeted therapies are available for the adenocarcinoma subtype.

The routine histological subtypization is based on Hematoxylin and Eosin (H&E) staining and five to six immunohistochemical reactions. Despite the low number of thin sections, the feasibility of high-quality nucleic acid isolation is endangered due to the size and consistency of the tissue core.

The above-mentioned aspect explains the need for new methods that can determine the origin of a single cell or group of cells without requiring immunohistochemical reactions, therefore, leaving more material for DNA or RNA isolation and consequent molecular pathological analysis. Infrared spectroscopy could do this and we aimed to build up classification models. We focused on the differential diagnostic application of FT-IR in the most frequent tumor types [6]. There are molecular, immunohistochemical markers to distinguish histological subtypes [7], however, these altogether small differences would not be detected by FT-IR since it is expected to reveal rather a spectral fingerprint characteristic of subtypes than a different quantitative compositional alteration on the level of specific proteins (e.g., p63, TTF1).

### Infrared Spectroscopy

The spectral range over 780 nm is called infrared, which is conventionally divided into near-, mid- and far-infrared (NIR, MIR, FIR, respectively). The wavelength range of NIR is defined from 780 to 2500 nm (12,820-4000 cm^−1^—since due to the dispersed Fourier-transform (FT) spectrophotometers the wavenumber is typically measured in units of cm^−1^), the wavelengths of MIR are between 2500 and 25,000 nm (4000-400 cm^−1^) and the FIR range is between 25 and 1000 μm (400-10 cm^−1^). The higher the wavenumber, the higher the energy of the light. NIR and MIR photons elevate the chemical bonds to higher energy levels, causing deformation motions (e.g., angular changes). The FIR light has lower energy so it can excite the rotation of the atoms in the bonds. The quick and non-destructive NIR and MIR spectroscopy techniques are mostly used for investigating biological systems, while FIR is less relevant from this point of view and is not applied because of the shallow energy level. The mid-infrared area includes the so-called fingerprint region (1800-400 cm^−1^) where lipids, protein, amide I/II and nucleic acid peaks are highly representative [8].

One of the earliest MIR spectroscopic applications was to determine the cis/trans conformation of lipids [9]. MIR techniques were developed to analyse ingredients in milk [10, 11] and wine [12, 13]. Both NIR and MIR techniques are widespread in the field of biological matrix analysis. The qualitative and quantitative application of NIR in the agro-food sector began in the 1960s. Plants, animal products and processed foods from these are samples of complex biological origin, containing various contents of water, proteins, lipids and carbohydrates. Infrared analytics of grains and cereal-based products became a widespread technology, with the main focus being on the changes in protein content [14] and quality during ripening [15], and the monitoring of milling [16]. Additionally, in the field of pharmacology chemical structure of drug compounds [17] and polymers such as hydrogels [18] are proved by MIR spectroscopy as well.

MIR photons have less energy, therefore, the spatial penetration is shorter whereas the signal-to-noise ratio of the MIR spectra is about two orders of magnitude higher than in the case of NIR. There are fewer medical applications of MIR methods than that of NIR applications. MIR optical fibre, however, have been commercially available since 2016, whereas earlier only laboratory tools existed [19–22]. In a study, the breast cancer imaging of 15 patients was carried out using mid- and long-wave infrared cameras [23]. In another study, urine samples from a small cohort of healthy women as well as female patients with gynaecological malignancies were investigated with MIR resulting in diagnoses with high accuracy [24]. The basic tissue processing of pathological specimens and an imaging protocol were created by Zahdi et al. [25]. A further study has highlighted the pitfalls and best practices of tissue preparation methods for FT-IR spectroscopic analysis [26]. The most common method is to use fluorescent dyes, however, there is also another approach which chooses marker-free FT-IR imaging as a tool with promising results on lung tumour subtyping [27]. Großerueschkamp et al. analysed fresh frozen samples using random forest algorithms. They set up a 3-level decision-making scheme and even at a more detailed level they were able to successfully recognize adenocarcinoma subtypes as well [27]. Gayoud et al. focused on squamous cell FFPE sample preneoplastic and neoplastic separations on 34 samples with PCA and PLS-DA tools [28]. Akalin et al. choose spectral pretreatment as first step using Mie scattering and extended multiplicative signal correction (EMSC) algorithms. They get rid of the low signal quality spectra gained from tissue microarrays (TMA) and then processed via hierarchic clustering and SVM [29]. Molecular expression patterns resulting also from mutated genes and proteins represent such a complexity that can not be expected to be precisely reflected by MIR spectra. MIR rather detects a fingerprint representative of the above-mentioned molecular complexity of a tumor. Mass spectrometry might focus either on specific molecular changes in the molecular composition or similarly detect a complex pattern. Spectral bands that can be assigned to chemical functions or to macromolcules are collected in a table published by Le Naour et al. [30]. The purpose of the present study was to differentiate lung tumor subtypes using label-free mid-infrared imaging.

## Materials and Methods

### Aluminium Coated Slides

Thin-film metal layers were deposited onto glass slides by vacuum evaporation to gain mid-infrared capable reflective surface. An electron-beam evaporation source was applied in a high-vacuum chamber, in which the glass slides were fastened onto the rotary sample holder. Aluminium was evaporated at 10^−4^ Pa for 20 min at an accelerating voltage of 7 kV and beam current of 200 mA, resulting in a layer thickness of *ca.* 150 nm.

### Lung Cancer Samples

A total of 30 FFPE lung cancer biopsies were selected from the archive of 2nd Department of Pathology Semmelweis University and Department of Pathology University of Pécs 10 for each subtype. 2 μm slides were cut from paraffin-embedded blocks and were deparaffinized two times 10 min xylene. The use of human FFPE samples was approved by the Hungarian Medical Research Council, Budapest, Hungary (no. 61303-2/2018/EKU). In the routine workflow the sample preparation takes 1 day after grossing.

### Infrared Imaging

**FIGURE 1:**
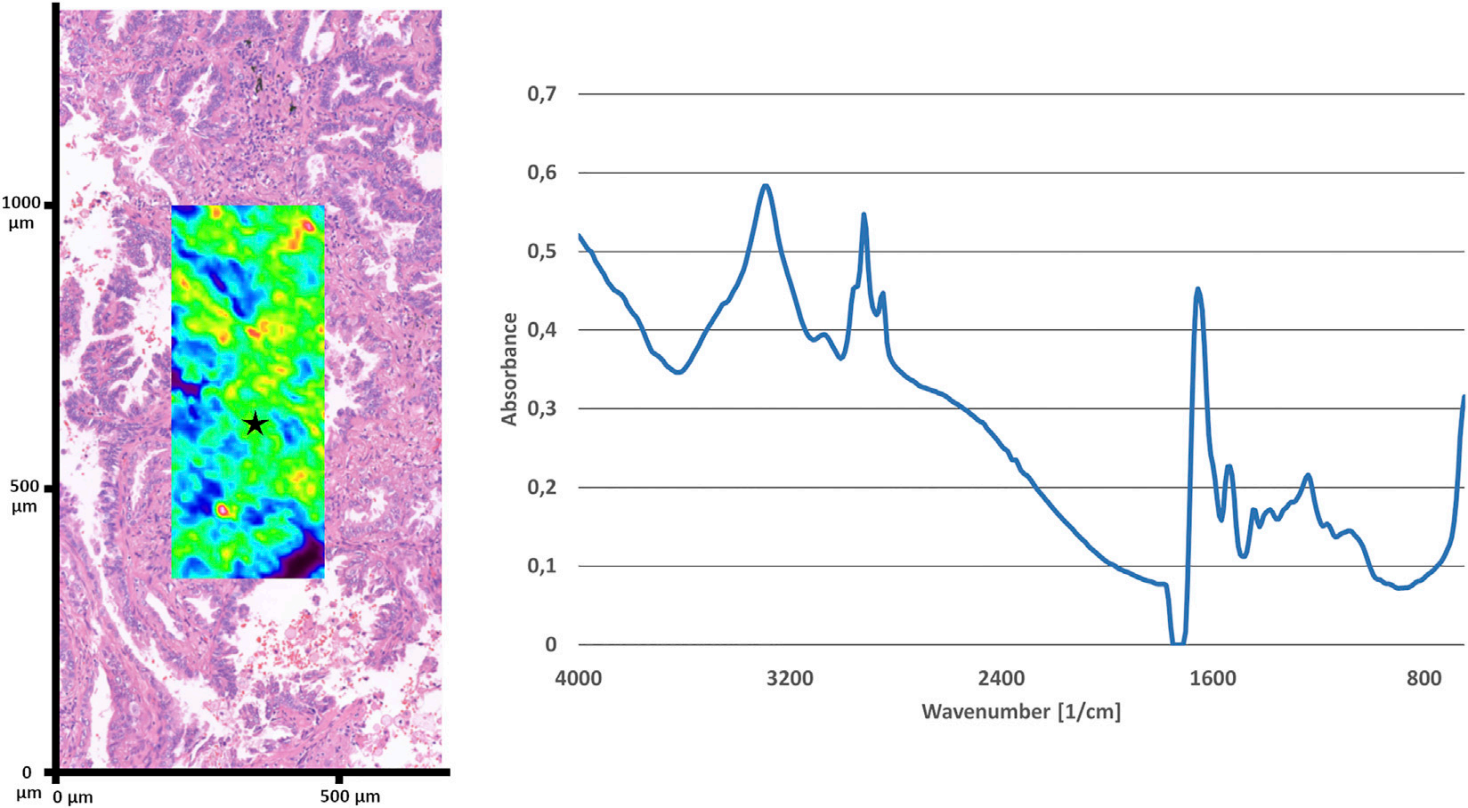
Representative H&E stained region of a LUAD and multicolor average absorbance infrared image overlayed over the identical area (on the left). Representative spectrum (4000-650 cm^−1^) of LUAD of the location marked with a star (on the right).

Fourier transform mid-infrared imaging was used for collecting spectra with transflection optical setup. The feasibility of this approach has been tested on the separation of ethanol fixed cell lines, this has been reported previously [31]. Spotlight 400 microscope (Perkin Elmer Inc., Waltham, MA, United States) was connected to Spectrum 400 spectrophotometer used for scanning images. The Mercury Cadmium Tellurite (MCT) detector collected spectra with 32 scans with a resolution of 16 cm^−1^ and data interval of 8 cm^−1^ were recorded for each spectrum in the mid-infrared wavelength range between 4000 and 648 nm. The 250 μm × 600 μm images were scanned (Figure 1) by pixel size 6.25 μm × 6.25 μm. A single image contained 40 × 96 pixels and resulted in 3,840 spectra on a 0.15 mm^2^ area. The acquisition time for each selected area was 46 min.

### Data Processing

#### Atmospheric Correction and Noise Reduction

The acquired images were treated with two built-in algorithms of the SpectrumIMAGE R1.6.5.0396 software (Perkin Elmer Inc., Waltham, Massachusetts, United States) for atmospheric correction and noise reduction. The atmospheric CO_2_/H_2_O suppression by the least square fitting of the algorithm affected the atmospheric correction of the spectra. (Patent No.: US 6,518,753 B1) The noise reduction was based on a 20-factor principal component analysis. Since the noise has lower weights, the 20-factor-based reconstructed spectrum is noise reduced. This method does not lead to the broadening of the spectrum peaks, unlike smoothing.

From the whole spectrum the so-called fingerprint subregion was analysed. In case of the fingerprint range of measured IR spectra there are 145 wavenumbers (1800-648 cm^−1^ wavenumber range) considered as variables. According to a large amount of data, it is difficult to interpret the data cube, therefore some information may partly stay hidden.

#### Support Vector Machine

Classification by SVM is a method based on statistical learning. The essence of SVM is to determine the hyperplane with the maximum margin for linearly separable data. The reason for the maximum margin is that decision boundaries with a large margin tend to have better generalization error than those with a small margin. The method has a wide range of applications in statistical analysis in almost all disciplines. The main advantage of the method is that it can be extended to non-linear data sets using kernel functions. In order to perform the analysis, the algorithm must be trained on a part of the data set, which contains a category variable, and then validated on the other part of the data. Once these operations have been performed, the accuracy of the method can be deduced from the results. Another advantage of the support vector machine is that it has good generalisation capabilities and can be easily applied to multidimensional data. The confusion matrix summarizes the prediction results on the classification problem. Unscrambler X 10.4 (CAMO Software AS, Oslo, Norway) software was applied to perform the SVMs.

#### Linear Discriminant Analysis

LDA is a method for separating two or more classes by considering several quantitative variables simultaneously. A prerequisite for its application is that objects are already divided into classes. We must, therefore, be familiar with the classes that are identified by this classifying variable.

LDA is a classification method in which n-dimensional patterns are transformed into an m-dimensional space (m < n) by linear transformation. Consequently, samples from the same class will be located close to each other, while samples from different classes will be located far in space. The method is a supervised classification method, unlike the unsupervised, e.g., Cluster and Principal component analyses. The purpose of LDA is to determine the best fitting parameters for grouping the samples in the constructed model. The already constructed model can be used to project unknown samples. LDA is an uncomplicated method to use and is approximated on Bayes’ formula [32]. The projected results can be also put in a confusion matrix as described earlier.

Unscrambler X 10.4 (CAMO Software AS, Oslo, Norway) software was applied to perform the classifications.

## Results

**FIGURE 2:**
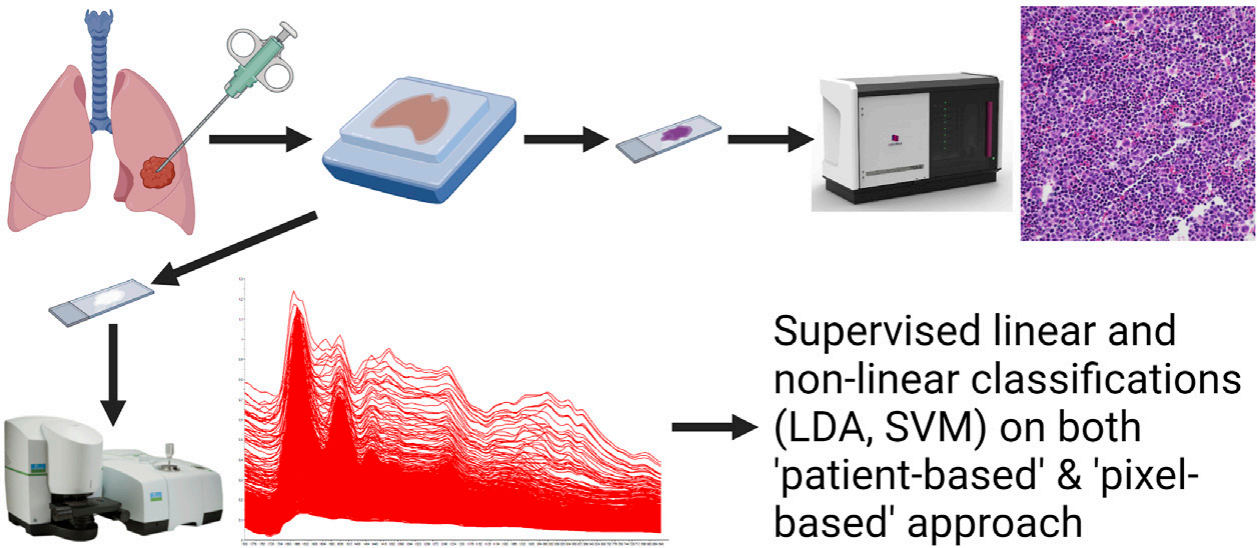
The scheme of experimental setup.

10-10 specimens were selected from each of the three histological subtypes (Table 1). Cases 2 and 6 are resected tumours from the same patient operated on two different locations at different timepoints. After identifying the tumorous region on the H&E slides, the consecutive slides’ parallel area was imaged on the aluminium coated slides using the infrared microscope. The scheme of technology is visualized by a graphical workflow (Figure 2). The acquired spectra were collected and treated with atmospheric correction and noise reduction.

**TABLE 1:** Clinicopathological features of the patients.

Case	Subtype	Age	Sex	Specimen type	Stage
1	SQ	54	Male	Resection	T2bNxMx
2	LUAD	48	Male	Resection	T2aNxMx
3	SQ	59	Male	Biopsy	Not applicable
4	SQ	67	Male	Biopsy	Not applicable
5	LUAD	57	Male	Resection	Not applicable
6	LUAD	48	Male	Resection	T3NxMx
7	SCLC	59	Male	Biopsy	T2N2M1
8	SQ	66	Female	Biopsy	T3N1M0
9	SCLC	51	Male	Resection	T2aN0Mx
10	SCLC	65	Male	Biopsy	T4N1M1
11	SQ	68	Male	Resection	T2aN0M0
12	SCLC	71	Male	Biopsy	T4N2M1
13	SQ	75	Male	Resection	T2bN0M0
14	SCLC	59	Female	Biopsy	T4N2M1b
15	LUAD	65	Female	Biopsy	T4N3Mx
16	SCLC	72	Female	Biopsy	T3N1Mx
17	LUAD	79	Female	Resection	T1aNxMx
18	SQ	65	Male	Biopsy	T3N0M0
19	SQ	60	Male	Resection	T1bNxMx
20	LUAD	78	Female	Resection	T2aNxMx
21	LUAD	63	Male	Resection	T1cN0Mx
22	LUAD	63	Female	Resection	T2aN2Mx
23	SCLC	57	Female	Resection	T2aN0Mx
24	LUAD	65	Female	Biopsy	TaN3M1c
25	SCLC	52	Male	Biopsy	T4N3M0
26	SCLC	64	Female	Biopsy	Not applicable
27	SCLC	69	Female	Biopsy	Not applicable
28	SQ	67	Male	Resection	T2bN0Mx
29	SQ	71	Male	Resection	T2aN0Mx
30	LUAD	58	Male	Resection	T2aNxMx

The spectra of the acquired images were put into a table, from which every second spectra were selected and used as a training set. A total of 5 analyses were performed using three LDA (using linear, quadratic and Mahalanobis distances) and two SVM (linear nu-SVC SVM and linear C-SVC SVM settings) models. We ran the other part of the datasets on these models and the methods predicted the histological subtype.

Two approaches were used to examine the data. We examined both “patient-based” and “pixel-based” analyses. The “patient-based” classifications are shown first. This approach is based on majority projection, therefore, the lowest recommended cut-off value is 50%. Each spectrum was classified individually by the five above-mentioned mathematical models. We have sorted the results by sample into bias matrices. The proportion of correctly classified spectra from each patient sample (3,840 spectra per sample) was considered as the decisive factor for the correct classification. The different cut-off values were compared in order to rank the accuracy of the models (Table 2).

**TABLE 2:** Accuracy of the prediction models using different cut-off values.

Models cut-off	Linear nu-SVC SVM	Linear C-SVC SVM	Linear LDA	Quadratic LDA	Mahalanobis LDA
50%					
SQ	90% (9/10)	100% (10/10)	70% (7/10)	100% (10/10)	0% (0/10)
SCLC	90% (9/10)	100% (10/10)	50% (5/10)	80% (8/10)	0% (0/10)
LUAD	10% (1/10)	100% (10/10)	20% (2/10)	20% (2/10)	100% (10/10)
60%					
SQ	80% (8/10)	80% (8/10)	60% (6/10)	100% (10/10)	0% (0/10)
SCLC	90% (9/10)	90% (9/10)	40% (4/10)	70% (7/10)	0% (0/10)
LUAD	0% (0/10)	80% (8/10)	20% (1/10)	20% (2/10)	100% (10/10)
70%					
SQ	50% (5/10)	80% (8/10)	50% (5/10)	90% (9/10)	0% (0/10)
SCLC	90% (9/10)	90% (9/10)	40% (4/10)	60% (6/10)	0% (0/10)
LUAD	0% (0/10)	70% (7/10)	20% (2/10)	20% (2/10)	100% (10/10)
80%					
SQ	20% (2/10)	70% (7/10)	50% (5/10)	90% (9/10)	0% (0/10)
SCLC	80% (8/10)	80% (8/10)	40% (4/10)	50% (5/10)	0% (0/10)
LUAD	0% (0/10)	60% (6/10)	20% (2/10)	20% (2/10)	100% (10/10)
90%					
SQ	10% (1/10)	20% (2/10)	40% (4/10)	70% (7/10)	0% (0/10)
SCLC	40% (4/10)	70% (7/10)	40% (4/10)	30% (3/10)	0% (0/10)
LUAD	0% (0/10)	60% (6/10)	20% (2/10)	20% (2/10)	100% (10/10)
95%					
SQ	0% (0/10)	20% (2/10)	40% (4/10)	30% (3/10)	0% (0/10)
SCLC	40% (4/10)	60% (6/10)	10% (1/10)	20% (2/10)	0% (0/10)
LUAD	0% (0/10)	40% (4/10)	20% (2/10)	20% (2/10)	100% (10/10)

Linear C-SVC SVM model with a 50% cut-off was the most successful regarding the separation of the subtypes. For interpretation of the table selecting e.g., 70% cut-off value and linear C-SVC SVM one could see that the cut-off value was not achieved in only 6 out of 30 cases: 2 samples in SQ, 1 SCLC and 3 samples in LUAD. However, these cases were also correctly predicted if we classify the samples based on the 50% cut-off value. Of course, the fine-tuning of the above-described method could improve the other models as well. It might be worthy of testing lower than 50% cut-off values, however, this approach would certainly require bigger cohorts and further testing of mixed differentiation such as adenosquamous carcinomas which feature both adenocarcinoma and squamous cell carcinoma subtypes.

By examining these five models on a pixel basis, we can obtain cumulative data for each subtype based on the prediction of each spectrum. The performance of the five models can be compared in terms of sensitivity, specificity, positive predictive value (ppv) and negative predictive value (npv) characteristics. Overall, consistent with the patient-based approach, the linear C-SVC SVM model proved to be the best again with sensitivity ranging from 81.645% to 88.885% and specificity from 90.484% to 94.784% regarding histological subtypes (Table 3). Quadratic LDA model achieved higher sensitivity for determining SQ predictions compared to linear C-SVC SVM but with lower specificity. Similarly, for spectra of LUAD samples, quadratic LDA gave 99% specificity but lagged behind SVM in sensitivity. The quadratic LDA has a 95.107% ppv for LUAD which is better than the 82.885% of the C-SVC SVM performance.

**TABLE 3:** The performance of the five models.

	SQ	LUAD	SCLC
Linear nu-SVC SVM
Sensitivity	71.290%	8.261%	84.192%
Specificity	51.706%	92.294%	87.872%
Ppv	42.465%	34.895%	77.633%
Npv	78.270%	66.800%	91.747%
Linear C-SVC SVM			
Sensitivity	81.645%	82.890%	88.885%
Specificity	90.484%	91.442%	94.784%
Ppv	81.096%	82.885%	89.495%
Npv	90.791%	91.445%	94.461%
Linear LDA			
Sensitivity	70.900%	20.001%	50.878%
Specificity	50.469%	100.000%	70.420%
Ppv	41.715%	100.000%	46.237%
Npv	77.622%	71.429%	74.141%
Quadratic LDA			
Sensitivity	91.562%	23.387%	68.405%
Specificity	58.896%	99.398%	83.382%
Ppv	52.692%	95.107%	67.300%
Npv	93.315%	72.182%	84.072%
Mahalanobis LDA			
Sensitivity	0.005%	100.000%	0.010%
Specificity	100.000%	0.008%	100.000%
Ppv	100.000%	33.335%	100.000%
Npv	66.668%	100.000%	66.669%

The classification by Mahalanobis LDA was completely wrong. The model considered virtually all spectra as adenocarcinomas. That is why a 100% sensitivity was obtained for the spectra of the LUAD samples, but the specificity is 0.008%. The SQ and SCLC specificities gave a vain value of 100% since the sensitivity values are close to zero.

## Discussion

In this paper, we present a marker-free and automated diagnostic FT-IR imaging-based tool for pathological decision support. Classification of histologically significant lung tumor subtypes was achieved. We also highlighted the differences between five multivariate data analysis models.

The accuracy of these models was calculated using several cut-off parameters. The higher the accuracy the lower the cut-off in general. A strong bias was observed regarding the Mahalanobis model because every sample was predicted into one class. The best separation was reached by Linear C-SVC SVM model combined with 50% cut-off value according to our findings. Further optimization of the cut-off value would require a larger cohort. The pixel-based predictions also proved to be successful. Overall the C-SVC SVM performed better than the other 4 models.

The discriminative power of Linear C-SVC SVM method outperformed the others, however, certain individual statistical metrics of the other methods—such as sensitivity, specificity, ppv, npv—were better regarding the histological subtypes. Experienced pathologists in their routine activity combine the advantages of several approaches, therefore, the above-mentioned parameters are excellent [33]. The Linear C-SVC SVM sensitivity and specificity values are in a comparable range with pathology-associated image analysis tools such as the PAPNET for cervical smear [34]. The infrared spectral analysis has a promising perspective to develop this method to assist intraoperative decision-making similar to mass spectrometry-assisted tools. The correct tissue classification by mass spectrometry was characterized by high accuracy with a sensitivity of 90.5% and specificity of 89.7% which is comparable with our method [35].

These approaches promise reproducibility, objectivity, and higher accuracy compared to current methodologies for lung tumor diagnostics. There is a growing need for personalization in medicine which requires a fast and accurate way of differential diagnosis. The approach has yet to be validated on a larger scale.

Altogether, the overall training time using half of the spectra for different models took between 2 and 12 h. However, the prediction time using these models was tremendously shortened: 5–10 min. The major delay in the current routine workflow of pathology is the sample preparation which needs 1 day after grossing. Based on the H&E image the infrared acquisition time of the selected area takes only 46 min. Analysis of native surgical specimens would reduce diagnostic intervals and enable on-site measurements. An intraoperative approach could also be executed with different detection e.g., Raman spectroscopy works better in an aqueous medium like unfixed, on-site specimens.

These *in vitro* approaches might serve as the basis to develop a dye-free intraoperative technique to facilitate surgical decision-making [27, 36]. Altogether, our *in vitro* results project the feasibility of infrared imaging to identify different cancer subtypes. Optional other ancillary methods would be the detection of tumor type specific tumor-associated DNA, however, there are no clinically reliable markers available so far.

The infrared technique would have an advantage as non-destructive and even on-site. We developed our method on histologically verified tissue sections. Long-standing problem is the adequacy of the sampling for the diagnostic needs. The oncoteam decision-making requires steadily increasing tissue-related diagnostic information. Certain genetic data need next generation sequencing, the other histological, immunohistochemical or fluorescent *in situ* hybridization information. Infrared spectroscopy might provide necessary data from less or minimal amounts of tissue. That means that biopsy material will be saved for other necessary methods, therefore, less or minimal invasive sampling would be enough [37].

The selection of the proper cut-off value secures the specificity of the analysis. Therefore, tumors featuring areas of different histological subtypes such as adenosquamous carcinomas of the lung could be used to test the feasibility of possibly lower cut-off values of the methods. This could improve the power of separation. Adenosquamous carcinomas are a rare mixed differentiation subtype of non–small cell carcinoma of the lung, constituting 0.4%–4% of cases. p63 IHC reaction is the tool to identify the squamous component. The lowest cut-off value could be determined on a large number of mixed entities.

Infrared imaging might be suitable to identify the lung cancer subtypes, therefore, fewer slides would be necessary for IHC and more tissue would be preserved for molecular pathology to select potential novel therapies. Larger datasets must be analyzed to further support our results.

In our study, label-free mid-infrared imaging was used to acquire spectra from three lung cancer subtypes. LDAs and SVMs were performed on all the investigated subgroups in the fingerprint mid-infrared region. Based on our results SVM models performed better although spectral pretreatments might further increase the accuracy, therefore, it could be an additional option. We successfully demonstrated the feasibility of our infrared method to separate cancer subtypes types by their label-free mid-infrared spectra with highlighted range. In conclusion, our data suggest the usage of transflective optical set and 1800-648 cm^−1^ spectral range to gain spectra.

## Data Availability

The raw data supporting the conclusion of this article will be made available by the authors, without undue reservation.
